# Assessing public forecasts to encourage accountability: The case of MIT’s *Technology Review*

**DOI:** 10.1371/journal.pone.0183038

**Published:** 2017-08-10

**Authors:** Jeffrey Funk

**Affiliations:** Self-Employed, Singapore, Singapore; University of Illinois at Urbana-Champaign, UNITED STATES

## Abstract

Although high degrees of reliability have been found for many types of forecasts purportedly due to the existence of accountability, public forecasts of technology are rarely assessed and continue to have a poor reputation. This paper’s analysis of forecasts made by MIT’s *Technology Review* provides a rare assessment and thus a means to encourage accountability. It first shows that few of the predicted “breakthrough technologies” currently have large markets. Only four have sales greater than $10 billion while eight technologies not predicted by *Technology Review* have sales greater than $10 billion including three with greater than $100 billion and one other with greater than $50 billion. Second, possible reasons for these poor forecasts are then discussed including an over emphasis on the science-based process of technology change, sometimes called the linear model of innovation. Third, this paper describes a different model of technology change, one that is widely used by private companies and that explains the emergence of those technologies that have greater than $10 billion in sales. Fourth, technology change and forecasts are discussed in terms of cognitive biases and mental models.

## 1. Introduction

Many types of forecasts have a high degree of reliability in terms of confidence levels and calibration [1]. Research has found that meteorologists [2], bridge players [3], and others [4][5][6][7] are well calibrated in terms of confidence. Similarly, a recent study of intelligence forecasters found that their forecasts explained 76% of the variance in geopolitical outcomes [8], a much better outcome than previous research [9]. The better forecasts are purportedly due to greater accountability [8].

Accountability is also an important part of forecasts made by private companies in a market economy. Private companies must make choices, many of these choices implicitly involve forecasts [8][1], and investors will punish private companies for bad choices [10]. Some organizational choices involve technologies and these choices also imply forecasts. Choosing a new technology implies a positive forecast while sticking with an old one implies a negative forecast. Similar arguments can be made for R&D budgets, investment plans, and product introductions where choices about specific technologies imply forecasts about them [11][12].

Investors reward companies that make good choices, i.e., forecasts, about new technologies. Apple’s high market capitalization reflects its successful introduction of MP3 players, smart phones, and tablet computers, all of which imply successful forecasts. Similar conclusions can be drawn from Amazon’s entry into eBooks, eReaders, and cloud computing, Facebook’s entry into social networking, video advertisements, and smart phone apps, and the evolution of IBM’s product lines over the last 100 years. Furthermore, some managers make better forecasts than do other managers because they can “look forward and reason back” [13] in order to identify new technologies and develop better strategies for them. David Yoffie and Michael Cusumano [13] concluded that doing this is a critical skill for managers such as Bill Gates, Steve Jobs, and Andy Grove. Throughout their careers, these three managers could look forward and identify trends, including necessary changes in products and services, and then reason back to develop appropriate strategies for the products and services.

On the other hand, public forecasts of technology have been heavily criticized [14][15] [16] with some exceptions [17], in spite of their clear importance. Decision makers in governments, universities, and other public organizations need access to good forecasts to make good decisions about new technologies. Governments must allocate funds to R&D [11][12], universities must choose which technologies to pursue, and cities must determine how to proceed with new infrastructure such as smart cities. This suggests that better technology forecasting is needed and a first step is an assessment of existing forecasts to provide more accountability, to understand the cognitive biases associated with public technology forecasts, and to propose better ways to make forecasts.

Cognitive biases are a major reason for poor forecasts and poor decision making in general. People have biases partly because they use heuristics to deal with a complicated world [18] and they are often over confident and miscalibrate their degree of confidence [19]. For example, people assess the relative importance of issues, including new technologies, by the ease of retrieving them from memory [20], this causes them to be optimistic about technologies that are regularly discussed by their peers or the mass media. Others may be biased towards a single factor and thus focus too much on this factor, rather than consider a wide variety of factors when forecasting technology or something else [1]. These biases often become stronger in groups in which there is strong pressure for conformity [15] [16] [21].

This paper assesses predictions of breakthrough technologies made by MIT’s *Technology Review* between 2001 and 2005 and it explains the (poor) forecasts in terms of cognitive biases particularly those associated with technology change. The next section summarizes the sources and methods of analyzing the data. The third section presents the current sizes of the markets for the technologies predicted and missed by MIT’s *Technology Review*. The fourth section interprets these results and possible reasons for the poor forecasts including an over emphasis on the science-based process of technology change, sometimes called the linear model of innovation. The over emphasis on the science-based process of technology change is a form of cognitive bias that comes from an emphasis on science in universities. The fifth section discusses an alternative model of technology change that is widely used by private companies, that explains the successful predictions by *Technology Review* and those missed by *Technology Review*, and that is consistent with one relatively successful technology forecast [17][22]. The sixth section discusses the theoretical and practical implications of searching for commercially viable technologies including cognitive biases, mental models and technological choices, including those for smart cities.

## 2. Methods

Predicted breakthrough technologies were acquired from MIT’s *Technology Review*, which provides news and analysis of new technologies. In the words of *Technology Review*, “the mission of (this publication) is to equip its audiences with the intelligence to understand a world shaped by technology” [23]. It does this with regular articles on technology, suppliers of technology, and to a lesser extent with forecasts of breakthrough technologies each year.

*Technology Review* chose 10 technologies in 2001 as predicted breakthrough technologies, and each year since 2003, most of which are related to electrical, computer, and bio-engineering. Or in its words, “We have chosen 10 emerging areas of technology that will soon have a profound impact on the economy and on how we live and work” [24] and this definition continues to be used. The predictions included a one-page description of the technology, which included the underlying science and its leading university researchers. These predictions were based on the editors’ discussions with experts in various technologies. Or in the words of *Technology Review*, these predictions were based on the “educated predictions of our editors (made in consultation with some of the technology’s top experts)” [24]. The emphasis on university researchers in the one-page descriptions suggests that these experts are university professors and researchers.

The success of these predictions is assessed by analyzing the current size of the markets. For breakthrough technologies to have a “profound impact on the economy and on how we live and work,” successful ones should have large market sizes. One definition of “breakthrough innovation” also emphasize market size, but uses the term consumption pattern; the full definition is “novel, unique, or state-of-the-art technological advances in a product category that significantly alter the consumption patterns of a market” [25]. The first half of this definition sounds like large changes in concepts and architectures, which is the standard definition of radical innovations [26], while the second half sounds like market size.

The current market sizes for the predictions made in 2001, 2003, 2004, and 2005 were analyzed to determine the impact that the predicted breakthrough technologies are having on the economy. Market sales data were gathered from market forecasting companies that provide a combination of historical and forecasted data for all applications of the technology. This paper’s analysis was careful to avoid forecasted data and gather only historical data usually from summaries of the reports. These forecasting companies include BCC Research, Research and Markets, Markets and Markets, Gartner, and Global Industry Analysts. After organizing the predictions by market size, this paper’s analysis then checked whether the market sizes were significantly higher for the older than newer predictions and thus whether markets for the newer predictions might grow rapidly in the near future.

The summaries of the market reports were found by using Google’s search engine. The name of the technology along with the terms “market size” and “sales” were used in the search engine. If the search results did not reveal any links to market reports, the names of the technologies were modified to increase the chances of finding a relevant report. The full list of search terms is shown in Appendix A in S1 File. These terms were found in *Technology Review’s* one-page descriptions and benefited from the author’s knowledge of technologies. For example, although *Technology Review* used the term “power grid control” in its predictions of a breakthrough technology, searching for the term “smart grid” found much more data and this is an example of a successful prediction by *Technology Review*. Similarly, the term “big data” was searched for in place of “data mining” and this also resulted in much more information being found. In all cases, the market size data depended on the market reports.

For the biology-related technologies, the analysis partly relied on input from a bio-engineering colleague. He provided help on the definitions of the technologies, their descriptions and compositions, the terms to use in a Google search for market reports, and whether the market sizes found in the reports made sense.

In general, when definitions were uncertain, this paper’s analysis either erred on the side of larger market sizes or excluded the technology from the analysis. The first criteria for excluding a technology was whether market data could be found. If no data could be found, the question was whether the size of the market was actually zero or because the term is not specific enough for market report companies to define products, services, and the market sizes for them. This paper chose to error on the latter side and attempted to understand if a technological term might be too broad for market research companies to gather and present data.

One type of evidence of an overly broad term is that the technology had been used long before the *Technology Review* forecast was done. For example, the terms mechatronics, software assurance, enviromatics, and robot design refer to broad sets of techniques that existed long before *Technology Review* made its forecasts and both the terms and their descriptions are not specific enough to distinguish between the old techniques and any new ones. Electronics started replacing electromechanical controls in the 1980s and thus mechatronics has existed for decades. Software assurance has been done since the early years (1950s and 1960s) of the software industry. Sensors (e.g., temperature, pressure) have been used to monitor our environment for centuries and thus enviromatics has existed for just as long. Robots and other mechanical products have been designed with computers since the 1980s and thus the term robot design is far too vague to define products and services.

Even if the term had not been used for decades, it still might be too broad for relevant market reports to exist. For example, relevant market reports did not emerge from a search of “universal translation” or the terms used in *Technology Review*’s one page-description; these terms include mathematical models and natural language processing. Relevant market reports did not emerge from a search of “Bayesian machine learning” or other terms used by *Technology Review* such as “probabilistic approaches” in combination with “computer programming.” Relevant market reports also did not emerge from a search of “untangling code” or other terms used by *Technology Review* such as “aspect oriented,” “adaptive programming,” or “subjective programming.” These terms do not have the specificity sufficient to define products and services and thus for market forecasting companies to estimate their market size.

Successful breakthrough technologies that were not predicted by *Technology Review* were also analyzed to contrast their market sizes with those technologies chosen by *Technology Review*. This paper focused on technologies that had not achieved growth when Technology Review made its forecasts between 2001 and 2005 and whether the technology had more than $10 billion in revenues by 2015. If it had growth when the forecast was made, then there is probably little uncertainty about its success and thus it should not be considered a missed technology by *Technology Review*.

The length of time a technology was known or the degree to which it was known are not important issues. Forecasts usually include well-known technologies that have undergone research for many years. For example, Technology Review chose brain-machine interface, digital rights management and biometrics in 2001 and these technologies had been researched for more than 30 years before the predications were made in 2001. An even better example is nuclear fusion. First conceived as a power source in the 1920s and the most widely used design (Tokamak) was created in the 1950s, it still elicits optimism among many scientists and engineers and it should be considered by technology forecasts.

Potential technologies were found in the business press including the *Wall Street Journal* and the *Economist* and they are not meant to be a complete list of recently successful breakthrough technologies. The purpose is to demonstrate that *Technology Review* missed a greater number of successful technologies (i.e., breakthrough technologies) than they predicted. Sales data was collected for 2015 as is data on when a new technology began to achieve growth. As with the breakthrough technologies predicted by *Technology Review*, sales data were taken from market research reports and the reports were found using Google’s search engine. Information about whether there was market growth by 2003 was also found with Google’s search engine and this often involved assessing whether a technology was introduced by 2003 in the form that eventually succeeded. Introduction is a more stringent criterion than is growth since it comes after introduction. The year 2003 was chosen because it is halfway between 2001 and 2005.

## 3. Results

The technologies chosen as breakthrough technologies by MIT’s *Technology Review* between 2001 and 2005 are listed in Table 1. The market sizes for these technologies are summarized in Table 2 and the references for the market sizes are listed in Appendix B in S1 File. One predicted breakthrough (Data Mining) has greater than $100 billion in sales, three have between $10 and $49 billion) (power grid control, biometrics, distributed storage), one has sales between $5 and $10 Billion (micro-photonics), six have sales between $1 and $10 Billion, 8 have sales between $100 million and $1 Billion, and 14 have sales less than $100 million. Most of these sales are for the year 2015. For seven of the technologies (Mechatronics, Enviromatics, software assurance, universal translation, Bayesian machine learning, untangling code, bacterial factories), neither sales data nor discussion of them, as they were defined by *Technology Review* could be found. Thus, these seven were excluded from the analysis and it is concluded that the terms were too vague or broad in their scope.

**Table 1:** Breakthrough technologies predicted by MIT’s Technology Review between 2001 and 2005.

2001	2003	2004	2005
Brain-Machine Interface:	Wireless Sensor Networks	Universal Translation	Airborne Networks
Flexible Transistors	Injectable Tissue Engineering	Synthetic Biology	Quantum Wires
Data Mining	Nano Solar Cells	Nanowires	Silicon Photonics
Digital Rights Management	Mechatronics	T-Rays	Metabolomics
Biometrics	Grid computing	Distributed Storage	Magnetic-Resonance Force Microscopy
Natural Language Processing	Molecular imaging	RNAi Interference	Universal Memory
Microphotonics	Nanoprint lithography	Power Grid Control	Bacterial Factories
Untangling Code	Software assurance	Microfluidic Optical Fibers	Enviromatics
Robot Design	Glycomics	Bayesian Machine Learning	Cell-Phone Viruses
Microfluidics	Quantum cryptography	Personal Genomics	Biomechatronics

**Table 2:** Market sizes for predicted technologies.

Market Size	Technologies
>$100 Billion	(1) Data Mining (Big Data)
$50–$100 Billion	
$10–$50 Billion	(3) Power grid control (Smart Grids), Biometrics, Distributed Storage,
$5–$10 Billion	(2) Micro-photonics, RNAi Interference,
$1–$5 Billion	(6) Grid computing, Molecular imaging, Synthetic Biology, Digital Rights Management, Natural Language Processing, Microfluidics
$100 Million—$1 Billion	(7) Wireless Sensor Networks, Metabolomics, Flexible Transistors, Personal genomics, Quantum cryptograp3hy, Silicon Photonics, Brain-Machine Interface,
< $100 Million	(14), T-Rays, Quantum Wires, Universal Memory, Injectable Tissue Engineering, Nano Solar Cells, Nanowires, Microfluidic Optical Fibers, Airborne Networks, Magnetic-Resonance Force Microscopy, Cell-Phone Viruses, Robot Design, Glycomics, biomechatronics. Nanoprint lithography,
Too Broad to Analyze	(7) Mechatronics, Enviromatics, software assurance, universal translation, Bayesian machine learning, untangling code, bacterial factories

Sources: See Appendix B in S1 File for sources of data.

One question is whether the markets are larger for the earlier than the later predictions, and thus will the markets for the earlier predictions grow quickly in the next few years. The data shown in Table 3 suggests that this is partly the case. Three of the six largest markets and six of the 12 largest markets are for technologies that were predicted in 2001. Looking at the technologies with small markets, the number of technologies with market sizes under $1Billion increased from 4 in 2001 to 10 in 2003, 6 in 2004 and 10 in 2005. However, the numbers for 2001 and 2004 are not that different. Furthermore, even for 2001 and 2003, excluding the three predicted breakthroughs that had too vague or broad of meanings to gather data, 8 of the 17 remaining predicted breakthrough technologies have sales of less than $1 Billion and only three have sales greater than $5 Billion.

**Table 3:** Market sizes vs. year of predictions.

	2001	2003	2004	2005	
>$100B	1				>$100B
$10-49B	1		2		$10-49B
$5-10B	1		1		$5-10B
$1-5B	3	2	1		$1-5B
$100M-1B	2	2	1	2	$100M-1B
<$100M	1	4	3	6	<$100M
unknown	1	2	2	2	unknown

Perhaps more importantly, *Technology Review* missed many important technologies that were introduced after 2003, that currently have global market sizes greater than $10 billion, and that have never made *Technology Review’s* list of breakthrough technologies, even after 2005 (See Table 4). Smart phones had a market of $400 billion, cloud computing had $175 billion, Internet of Things had $130 billion, tablet computers had $60 billion, and four others had between $10 billion and $24 billion (social networking, fintech, eBooks, and wearable computing). These technologies have had a “profound impact on the economy and on how we live and work, which is the definition used by *Technology Review* to choose breakthrough technologies. Each of these missed technologies also involved large changes in the concepts and architectures and thus could be defined as radical [25] or breakthrough innovations [24].

**Table 4:** Successful technologies missed by MIT’s Technology Review.

Technology	First Introduced	Sources	Market Size (2015)	Sources
Smart Phones	iPhone in 2007	s://www.engadget.com/2007/11/21/kindle-sells-out-in-two-days/	$400 Billion	https://www.statista.com/statistics/237505/global-revenue-from-smartphones-since-2008/
Cloud Computing	Amazon from 2006	https://en.wikipedia.org/wiki/Cloud_computing	$175 billion	http://www.gartner.com/newsroom/id/3188817 (does not include storage)
Internet of Things	“IoT born between 2008, 2009”	http://www.cisco.com/c/dam/en_us/about/ac79/docs/innov/IoT_IBSG_0411FINAL.pdf	$130 billion	http://www.postscapes.com/internet-of-things-market-size/
Tablet Computers	First iPad, 2010	http://blog.seattlepi.com/microsoft/2010/03/22/the-ipad-tablet-pc-market-defined/	$60 billion	http://www.idc.com/getdoc.jsp?containerId=prUS25867215
Social Networking	Facebook, 2004	https://www.wsj.com/articles/SB118539991204578084	$23.6 billion	http://blog.hubspot.com/customers/2016-paid-social-media-advertising-almost-took-over-the-world
Fintech	P2P lending, 2005	http://fintechnews.sg/3518/crowdfunding/asias-top-7-peer-peer-lending-platforms/	$20 billion	http://investingnews.com/daily/tech-investing/fintech-investing/fintech-market-size-a-breakdown-of-the-basicssub/
eBooks	Kindle, 2007	https://www.engadget.com/2007/11/21/kindle-sells-out-in-two-days/	$14.5 billion (only U.S.)	http://newsbreaks.infotoday.com/NewsBreaks/Ebooks-in-2015-Trends-and-Forecasts-Part-1-101446.asp
Wearable Computing	Fitbit, 2007	https://www.wareable.com/fitbit/youre-fitbit-and-you-know-it-how-a-wooden-box-became-a-dollar-4-billion-company	$13.8 billion	http://www.postscapes.com/internet-of-things-market-size/

Interestingly, the only technologies related to smart phones that were chosen by *Technology Review* were cell-phone viruses in 2005 and ultra-private smart phones in 2014. This is although smart phones are currently mentioned almost daily by most business magazines such as the *Wall Street Journal* and the *Financial Times*. They have enabled many other businesses to emerge such as ride sharing, fintech, and mobile commerce, and their most profitable supplier (Apple) has had the highest market capitalization each year since 2012.

## 4. Interpretation

Why were MIT *Technology Review’s* forecasts so poor? They were much less accurate than recently analyzed strategic intelligence forecasts [8]. They were also not as accurate as the technology forecasts made by Herman Kahn and Anthony Wiener in 1967 [22] that were analyzed in a 2002 paper [17]. More than 40% of the predictions made by Kahn and Wiener were judged to have become successful while only four of the 40 (10%) predicted breakthroughs achieved more than $10 Billion in sales.

One possible explanation for the bad forecasts is lack of accountability, which has been shown to improve predictions in many ways [4][5][6][7]. Most recently, it was concluded that accountability was the key to strategic intelligence forecasts [8]. For *Technology Review’s* predictions, perhaps the long-time frames have made it difficult to analyze the market growth of the predicted breakthroughs and provide feedback to the predictors. If so, this paper can provide feedback.

A second possible explanation is that not enough time has passed and thus we cannot judge the predictions made by *Technology Review*. This begs the question of how long of a time frame should be used and of whether a time frame longer than 10 to 15 years, which was used in this paper, is useful. As a timeframe increases past ten years, the usefulness of a forecast declines. Presumably the forecasts are supposed to help make better decisions about the technologies to fund, study, and understand, and a time frame longer than 10 (or 15) years would be too long for most of us. Furthermore, the fact that *Technology Review* missed smart phones, cloud computing, Internet of Thingstablet computers, social networking, fintech, eBooks, and wearable computing is probably a bigger problem than the fact that the markets for *Technology Review’s* predictions are small. If forecasters can’t get things right in a short-time frame, how can they get things right in a longer-time frame? Using a longer time frame will not solve this problem.

A third possible explanation is that MIT’s *Technology Review* used a different definition of breakthrough technologies than this paper used to assess the forecasts. Perhaps its words, “a profound impact on the economy and on how we live and work” [22], which it still uses, refers to something different than large market size. Maybe “a profound effect” refers to the degree of change in the technologies, their indirect impact on other technologies, or the use of science of the technologies? This seems unlikely, however, for many reasons. The technologies missed by *Technology Review* also involved large changes in the technology and thus they would be defined as breakthrough innovations [25] or as radical innovations [26]. A large indirect impact would also have been picked up by the keyword search, and yet articles discussing this indirect impact were not found. And if *Technology Review* meant “breakthrough research,” “breakthrough science,” “breakthrough idea,” or something else, it should have used these words instead of “breakthrough technologies” and “profound impact on the economy.”

Bluntly speaking, the term “breakthrough technology” implies a product or service and the term “profound impact on the economy” implies a monetary measure of market size. Suggesting that *Technology Review* meant something different than they wrote implies that *Technology Review* has misled its readers, an accusation that this paper is by no means making. This paper assumes that *Technology Review* meant technologies and the market size of those technologies when it used the terms “breakthrough technologies” and profound impact on the economy.”

A fourth and more likely reason for the bad forecasts is cognitive biases. Since people assess the relative importance of issues, including new technologies, by the ease of retrieving them from memory [20], this causes them to be optimistic about technologies that are regularly discussed by their peers. This would cause a small circle of experts to bias the predictions towards the experts’ own areas of research and scientific disciplines. Since *Technology Review’s* predictions were based on the “educated predictions of our editors (made in consultation with some of the technology’s top experts)” [24] and each prediction included the names of the leading university researchers, this was likely the case.

To investigate the possibility of cognitive biases in more detail, the problem of technology forecasting can be reframed in terms of technology change. Models of technology change form the basis for how experts interpret technology change and thus the viability of new technologies. The predominant view of technology change is the science-based model of technology change, sometimes called the linear model of invention [27]. Advances in science—new explanations of natural or artificial phenomena—play an important role in this process is because they facilitate the creation and demonstration (i.e., invention) of new concepts [27] [28][29][30]. They also facilitate the development of new product and process designs [31] [32][33][34] that lead to improvements along cost and performance trajectories [35].

The importance of science to this model of technology change and the emphasis on science by U.S. science and engineering departments suggests that this model of technology change is an important part of the mental models in U.S. science and engineering faculties. This is particularly the case since the publication of Vannevar Bush’s *Science*, *the Endless Frontier* in 1945 and the creation of the National Science Foundation in 1947. Publishing papers particularly those that provide explanations of physical or scientific phenomena, i.e., science—has become the most important activities in U.S. universities [27][36]. The science-based model of technology change biases forecasts towards technologies that are reported in science and engineering journals and towards disciplines that exist in science and engineering schools. It also biases forecasts away from technologies that can be defined as new products and services such as smart phones, smart phone apps, new forms of electronic products, and Internet content.

Support for this interpretation comes from the names of the breakthrough technologies chosen by MIT’s *Technology Review*. Many of the breakthrough technologies sound more like research disciplines than products or services, and this is consistent with a hypothesis that a science-based process of technology change formed the basis for the predictions made by MIT’s *Technology Review*. For example, consider the following predicted breakthrough technologies: synthetic biology, universal memory, metabolomics, universal translation, glycomics, T-rays and bacterial factories. They sound more like research disciplines than products and services. Contrast the names of these predicted breakthrough technologies with successful ones missed by MIT such as smart phones, cloud computing, Internet of Things, tablet computers, social networking, fintech, and eBooks and the differences between products/services and research/scientific disciplines can be clearly seen.

A focus on the science-based process of technology change may also be a major reason why market data were not found for many of the predictions and thus seven of the technologies were excluded from the analysis. These seven are Mechatronics, Enviromatics, software assurance, universal translation, Bayesian machine learning, untangling code, and bacterial factories. As noted in the methods section, many of these terms refer to broad sets of techniques that existed long before *Technology Review* made its forecasts and thus they are more consistent with research disciplines than technologies that might form the basis for new products and services. The choice of these “terms”, i.e., technologies, provides further evidence of a poor forecast since good forecasts should include definable entities.

This overall interpretation of *Technology Review’s* poor forecasts is also very consistent with management research on incumbent failure. Incumbents fail because new technologies require a new form of capabilities [37], architectures [24], lead customers [38], and business models but the incumbents have trouble making these changes because the new capabilities, architectures, customers, and business models require a change in dominant logic or mental model [39]. A similar situation appears to exist with the predictions made by MIT’s *Technology Review*. MIT’s *Technology Review* and its circle of experts appear to believe in a certain model of technology change that is emphasized at universities. Universities emphasize the linear model of invention, one that depends on advances in science, because this is how the research by university faculty are evaluated. Researchers develop better explanations for physical and artificial phenomena as they and other researchers develop and improve technologies that partly benefit from these advances in science. Thus, it is only natural that MIT’s *Technology Review* emphasizes a science-based model of technology change and thus made the types of predictions that they made.

## 5. Rethinking technology change

The poor forecasts by MIT’s *Technology Review* and its likely emphasis on a science-based process of technology change suggests there might be a different process by which new technologies become economically feasible and monitoring this process might result in better predictions of breakthrough technologies. Such a model can be deduced from the literature on general purpose technologies [40][41][42][43][44]. GPTs (general purpose technologies) have a large impact on many economic sectors of which recently defined GPTs are primarily electronic components or electronic products/systems. Examples of the former include lasers and integrated circuits (ICs) and examples of the latter include computers and the Internet [40][41][42][43]. One reason computers have been defined as GPTs is because they have had a large impact on the productivity of higher-level systems [44][45], and economic growth [46][47][48]. The improvements in these computers are typically attributed to improvements in standard ICs such as microprocessors and memory by computer scientists [49], economists [41], and management scholars [50][51].

One reason these ICs and other electronic components are defined as GPTs is because they have experienced rapid improvements, typically over many decades. For example, often called Moore’s Law, the number of transistors per chip for microprocessors, the number of memory bits per flash memory and dynamic random access memory (DRAMs), and the number of pixels per camera, i.e., photo-sensor, chip have doubled every 18 to 24 months for many years, resulting in relatively constant annual rates of improvement of 30% to 40% per year [34] over the last 50 years. Although these rapid improvements have depended on advances in science [52], it is the impact of these better ICs, lasers, and other GPTs on the emergence of new products and services that is of interest to forecasters and to this paper.

The impact of these GPTs on higher level systems, products, and services suggests to many that monitoring this impact can help decision makers find commercially viable technologies. Variations of this model have been described by Silicon Valley practitioners [53][54][55] and some academics [56][57][58] in which high-tech managers such as Bill Gates, Steve Jobs and Andy Grove “look forward and reason back” [13]. This “Silicon Valley Model” explains the emergence of opportunities exploited by the Wall Street Journal’s billion-dollar startup club [59]. It is also consistent with a 2002 finding [17] that the highest success rates in Herman’s Kahn and Anthony Wiener’s well-known forecast in 1967 for 2000 [25] were for innovations with rapidly improving underlying technologies, most of which are defined as GPTs.

This model also explains the emergence of those *Technology Review* predictions that now have large markets. Although most of the predictions reflect the science-based model of technology change, the predictions that now have large markets benefited from improvements from electronic components and the Internet, as do the technologies missed by *Technology Review*. The former includes data mining (Big Data), power grid control (smart grids), biometrics, and distributed storage (cloud storage). The latter includes smart phones, cloud computing, Internet of Things, tablet computers, social networking, fintech, ebooks, and wearable computing.

Some of these technologies (biometrics, smart phones, tablet computers, and wearable computing) became economically feasible as rapid improvements in microprocessors (i.e., Moore’s Law), memory and displays occurred. More specifically, fast processors were needed to analyze finger prints, voices, and other biometric data. For electronic hardware such as phones, Apple’s app-based strategy became economically feasible as large amounts of inexpensive flash memory became available [55][60][61][62].

Other technologies (cloud computing, distributed storage, Big Data, fintech, smart grids, social networking, the Internet of Things, and ebooks) emerged as improvements in Internet speed and cost made them economically feasible. Most data processing and storage has moved from private data centers to the cloud with public clouds now providing most processing and storage [63]. Big Data services, such as those for fintech [64], benefit from the success of the cloud along with improvements in speed and cost of Internet services and the exploding amount of Internet data [65]. Social networking sites did not succeed until fast Internet speeds enabled Facebook to adjust a user’s connections each time they log on [66].

The economics of the Internet of Things and ebooks have depended on improvements in both the Internet and the relevant hardware. The IoT depends on inexpensive wireless modules, sensors, and services. eBooks depend on inexpensive and fast Internet services along with inexpensive eBook readers and tablet computers. The emergence of these technologies, both successful and missed predictions by MIT’s *Technology Review*, can be better explained by the Silicon Valley than the science-based model of technology change.

## 6. Discussion

Organizations must make choices and many of these choices involve forecasts. Because investors reward companies that make good choices, forecasts by private companies are probably much better than public forecasts. Yoffie and Cusumano [13] concluded that one reason for the success of Apple, Microsoft, and Intel is that they could make better forecasts than do other companies. They could “look forward and reason back” [13] to identify new technologies and develop better strategies for them.

On the other hand, public forecasts about new technologies have been largely criticized [14][15][16] and *Technology Review’s* forecasts were even worse than many of these forecasts. They were greatly inferior to those made by Herman Kahn and Anthony Wiener [25] in 1967 about the year 2000 that were analyzed in a 2002 paper [17]. More than 40% of the predictions made by Kahn and Wiener were judged to have become successful while only one of the 40 (2.5%) predicted breakthroughs achieved more than $10 Billion in sales. Furthermore, no technologies were identified as “missed” by Kahn and Wiener [17]. If companies made forecasts as bad as those made by MIT’s *Technology Review* (and acted on them), those companies would probably have experienced severe financial problems. This statement is not intended to criticize MIT or other universities but to emphasize that problems come from poor forecasts.

Practically speaking, city, state, and federal governments must also make choices about new technologies, particularly in complex systems [67][68]. Should they investigate science-based technologies such as hydrogen vehicles, superconducting transmission lines, mag-lev trains, synthetic food, fusion, and hyperloop, or should they investigate technologies that are emerging from the Silicon Valley process of technology change such as the Internet of Things, Big Data, ride sharing, driverless vehicles, drones, smart payment, mobile payments, online education, augmented reality, and virtual reality? Although the two sets of technologies are not directly comparable, the point is that cities make choices about which technologies to investigate, and these choices will be impacted by the process of technology change that they monitor.

Another way to think about the choices facing cities is to think of smart cities and the technologies related to them. One reason the term “smart cities” is now widely used [69][70] is because the technologies mentioned in the previous paragraph are emerging from the Silicon Valley process of technology change through rapid improvements in electronic components, the Internet, and smart phones. Understanding the distinction between the Silicon Valley and the science-based process of technology change can help cities make better decisions about the technologies to investigate and thus the policies to consider.

Future research should investigate the Silicon Valley process of technology change, its implications for decision makers, and the best way to monitor it and make forecasts. One hypothesis is that monitoring and forecasting this model probably requires a different set of people than were used by MIT’s *Technology Review*. Entrepreneurs, members of private firms and in particular Internet-based firms understand this process better than do university researchers. As noted above, university experts typically emphasize scientific and research disciplines rather than new products such as tablet computers or smart phones. University experts may not even currently perceive smart phones and tablet computers as important technologies, even though industry experts and students would.

The Silicon Valley process of technology change also requires forecasters to consider a wider number of factors than does the science-based model of technology change. While the names of science-based technologies can be taken from science and engineering journals or science and engineering research disciplines within universities, monitoring the Silicon Valley process of technology change requires forecasters to analyze the impact of rapidly improving technologies on the emergence of higher level products and services. The latter requires much more complex analysis than does the former partly because these products and services are not discussed in science and engineering journals.

This focus on the number of factors is consistent with the distinction between foxes and hedgehogs that is made by Tetlock and Gardner in their book *Superforecasting*: *The Art and Science of Prediction* [1] and that has been reported in Tetlock’s academic papers [4][5][9]. They argue that foxes make better forecasts because they focus on a larger number of factors than do hedgehogs. Hedgehogs make predictions based on what they believe are a few fundamental truths while foxes draw on diverse strands of evidence and ideas. Those who solely monitor advances in science might be called hedgehogs because they focus on a single issue, what is published in science and engineering journals, and they strongly believe in these journals. Those who monitor the Silicon Valley process of technology change might be called foxes because they draw on a diverse set of factors; these include improvements in various electronic components, computers, and the Internet and the impact these improvements have on the emergence of new products and services.

This leads to two testable hypotheses. Predictions based on the Silicon Valley model of technology change will be more accurate than those based on the science-based model of technology change. Second, predictions made by foxes will be more accurate than those made by hedgehogs. Future research should investigate these hypotheses and related issues further.

## 7. Conclusions

This paper does a rare assessment of a public technology forecast to encourage accountability and improve our understanding of technology change. It first shows that few of the breakthrough technologies predicted by MIT’s *Technology Review* between 2001 and 2005 currently have large markets. Only one of its predictions has sales greater than $100 billion and only three others have greater than $10 billion although other breakthroughs not predicted by Technology Review have sales greater than $100 billion (three), one between $50 and $99 billion, and four between $10 and $49 billion. Second, it shows evidence that Technology Review’s forecasts were largely based on a science-based process of technology change, sometimes called the linear model of innovation. Third, this paper describes a different model of technology change, one that is widely used by private companies and that explains the emergence of those technologies that have greater than $10 billion in sales.

## Supporting information

S1 File Containing Appendix A and Appendix B. (DOCX)
