# Supplementary material for: Assessing public forecasts to encourage accountability: The case of MIT’s Technology Review
Source: PLoS One. 2017 Aug 10;12(8):e0183038. doi: 10.1371/journal.pone.0183038 (PMC5552300; doi:10.1371/journal.pone.0183038)
Supplement: S1 File — (DOCX) [file pone.0183038.s001.docx]

Appendix A. Additional Search Terms Used to Find Market Reports

| Year | Technology | Additional search terms (different searches are separated by semi-colons) |
| --- | --- | --- |
| 2001 | Data mining | knowledge discovery in databases; web site logs; business analytics, Big Data |
|  | Robot design | computers design test configure robots |
| 2003 | Grid computing | Distributed grid computing |
| 2004 | T-rays | Terahertz rays |
|  | Distributed storage | Cloud storage |
|  | Power grid control | Smart grids |
|  | Personal genomics | DNA sequencing, 23andMe |
|  | Airborne Networks | Planes fly without ground controllers |
|  | Universal Translation | Mathematical models; natural language processing |
|  | Bayesian machine learning | Probabilistic approaches; computer programming |
| 2005 | Silicon photonics | Silicon lasers |
|  | Universal memory | Nanoelectronics; nantero |
|  | Bacterial factories | Metabolic engineering |
|  | Enviromatics | Sensors monitor environment |
|  | Cell-phone viruses | Cell phone virus software |
|  | Biomechatronics | Sensors, microprocessors, prosthetics |

Appendix B: Sources of market size data for Table 2

| Market Size | Technologies |
| --- | --- |
| >$100 B | Data mining <http://www.informationweek.com/big-data/big-data-analytics/> big-data-analytics-sales-will-reach-$187-billion-by-2019/d/d-id/1325631 |
| >$10 Billion | Power grid control (Smart Grids) [//www.whatech.com/market-research/energy/](https://www.whatech.com/market-research/energy/) 209349-global-smart-grid-global-from-2014-to-2020-scrutinized-in-new-research  Distributed Storage.  http://www.marketsandmarkets.com/PressReleases/cloud-storage.asp  Biometrics: [www.marketsandmarkets.com/PressReleases/biometric-technologies.asp](http://www.marketsandmarkets.com/PressReleases/biometric-technologies.asp) |
| $5 - $10 Billion | Micro-photonics: [http://www.bccresearch.com/market-research/photonics/ photonic-crystals-markets-technology-report-pho004b.html](http://www.bccresearch.com/market-research/photonics/%20photonic-crystals-markets-technology-report-pho004b.html)  RNAi Interference: [http://www.mordorintelligence.com/industry-reports/ global-rnai-technology-market-industry](http://www.mordorintelligence.com/industry-reports/%20global-rnai-technology-market-industry) |
| $1 - $5 Billion | Grid computing: <http://www.cisco.com/c/dam/en/us/solutions/collateral/> service-provider/service-provider-infrastructure-as-a-service/IaaS_BDM_WP.pdf  Molecular imaging <http://www.prnewswire.com/news-releases/global-markets-> and-technologies-for-molecular-imaging-devices-273673321.html  Synthetic Biology: [www.alliedmarketresearch.com/press-release/synthetic-](http://www.alliedmarketresearch.com/press-release/synthetic-) biology-market-is-expected-to-Reach-38-7-Billion-Globally-by-2020.html  Digital Rights Management <http://www.marketsandmarkets.com/PressReleases/> digital-rights-management.asp;  Natural Language Processing [www.marketsandmarkets.com/PressReleases/](http://www.marketsandmarkets.com/PressReleases/) natural-language-processing-nlp.asp;  Microfluidics: [www.marketsandmarkets.com/PressReleases/microfluidics.asp](http://www.marketsandmarkets.com/PressReleases/microfluidics.asp) |
| $100 Million - $1 Billion | Wireless Sensor Networks [http://www.businesswire.com/news/home/ 20160826005019/en/Industrial-Wireless-Sensor-Network-Market-Grow-Impressive](http://www.businesswire.com/news/home/%2020160826005019/en/Industrial-Wireless-Sensor-Network-Market-Grow-Impressive)  Metabolomics: www.marketsandmarkets.com/PressReleases/metabolomics-technology.asp  Flexible Transistors [www.idtechex.com/research/reports/printed-and-thin-film-](http://www.idtechex.com/research/reports/printed-and-thin-film-) transistors-tft-and-memory-2013-2023-forecasts-technologies-players-000358.asp  Personal Genomics: [http://cruwys.blogspot.sg/2015/01/what-is-current- size-of-consumer.html](http://cruwys.blogspot.sg/2015/01/what-is-current-%20size-of-consumer.html). <https://www.genome.gov/27565109/the-cost-of-> sequencing-a-human-genome/  Quantum cryptography: [www.slideshare.net/GlobalIndustryAnalystsInc/](http://www.slideshare.net/GlobalIndustryAnalystsInc/) quantum-cryptography-a-global-strategic-business-report  Silicon Photonics:  [www.marketsandmarkets.com/Market-Reports/silicon-photonics-116.html](http://www.marketsandmarkets.com/Market-Reports/silicon-photonics-116.html)  Brain Machine Interface  https://www.alliedmarketresearch.com/brain-computer-interfaces-market  andmarkets.com/Market-Reports/printed-electronics-market-197.html; |
| < $100 Million | T-Rays: [www.bccresearch.com/market-research/instrumentation-and- sensors/terahertz-radiation-technologies-markets-ias029b.html](http://www.bccresearch.com/market-research/instrumentation-and-%20sensors/terahertz-radiation-technologies-markets-ias029b.html)  No data was found for these technologies and the general discussions of these technologies on the Internet implied that the markets for them were almost zero. Quantum Wires, Universal Memory. Injectable Tissue Engineering, Nano Solar Cells, Nanowires, Microfluidic Optical Fibers, Airborne Networks, Magnetic-Resonance Force Microscopy, Cell-Phone Viruses, Robot Design, Glycomics |

These websites were last accessed on October 4 and 5, 2016
